# Breast Pumps and Mastitis in Breastfeeding Women: Clarifying the Relationship

**DOI:** 10.3389/fped.2022.856353

**Published:** 2022-06-10

**Authors:** Leon R. Mitoulas, Riccardo Davanzo

**Affiliations:** ^1^School of Molecular Sciences, The University of Western Australia, Perth, WA, Australia; ^2^Medela AG, Baar, Switzerland; ^3^Institute for Maternal and Child Health Institute, IRCCS “Burlo Garofolo”, Trieste, Italy

**Keywords:** mastitis, human milk, breastfeeding, breast pump, breast milk expression

## Abstract

Mastitis is a debilitating condition that can impact around 20% of mothers and is characterized by fever, flu-like symptoms and tender, swollen areas of the breasts. Despite the emerging evidence that breast milk dysbiosis is an underlying cause of mastitis, breast pumps have been implicated as a predisposing risk factor in the pathophysiology of mastitis in breastfeeding mothers. Previous studies have suggested that the use of a breast pump increases a mother's risk for developing mastitis, however, incidence rates of mastitis over the stages of lactation do not match breast pump usage rates. Furthermore, breast pumps, even when used at low vacuum, still promote some breast drainage, thus avoiding milk stasis, which is considered a key factor in the development of mastitis. As a consequence, these data suggest that the literature association of breast pumps with mastitis is more a case of reverse causation and not direct association. Moreover, it is important to note that breast pumps are actually a part of the conservative management of mastitis. In combination, these data show that the breast pump should not be considered a driver in the pathophysiology of mastitis in women.

## Introduction

**Figure 1:**
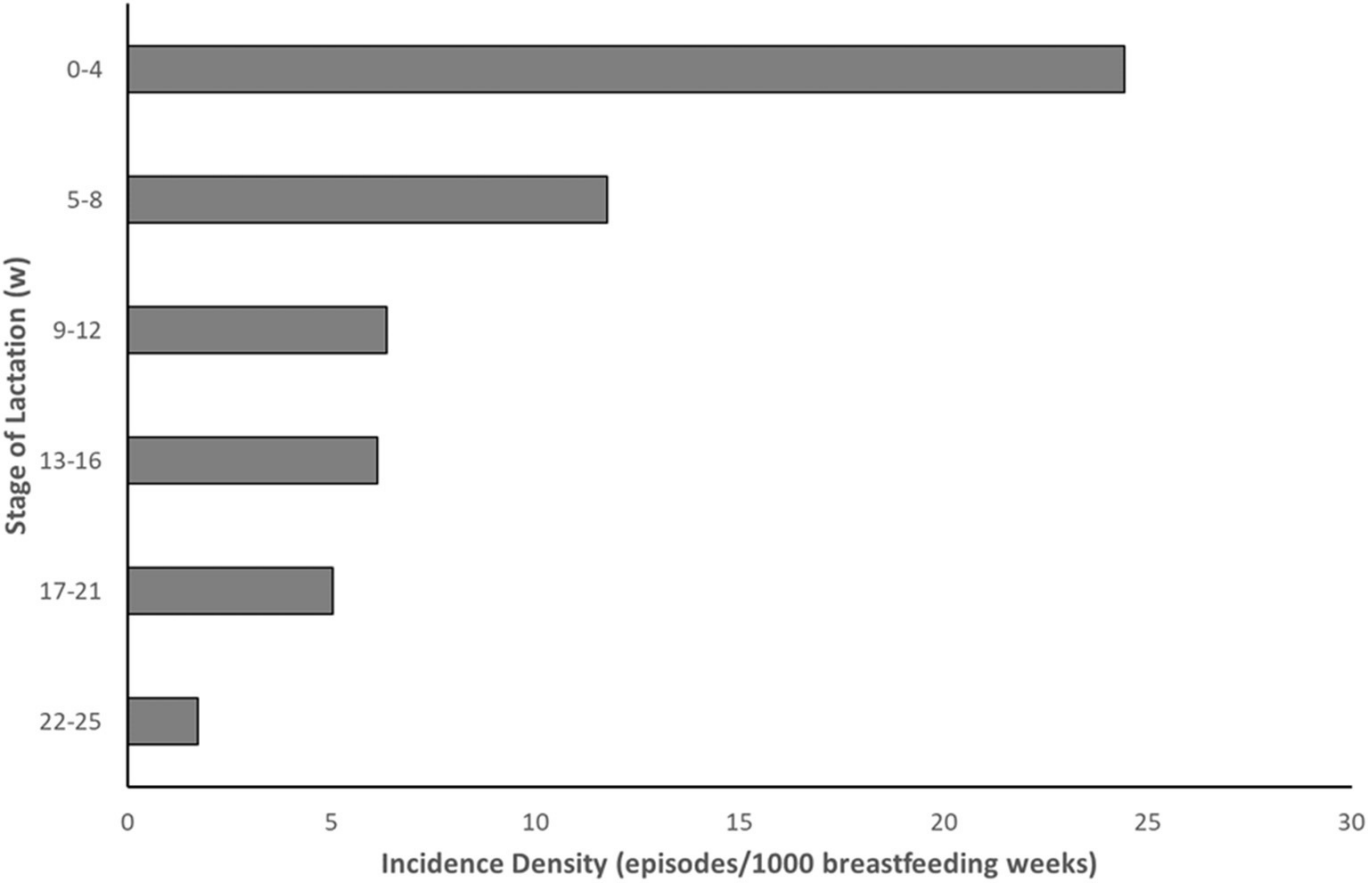
Incidence of mastitis over the course of lactation [Data adapted from Wilson et al. (3)].

Lactational mastitis, characterized by fever, flu-like symptoms and tender, swollen areas of the breasts (1), is a debilitating condition experienced by many breastfeeding mothers (2). The incidence rate of mastitis varies throughout the literature, with some texts suggesting up to 33% of mothers will experience mastitis (2). Indeed, the variability exhibited in the literature may result from the changing incidence rate associated with stage of lactation and the lack of standardization in the timing of studies (Figure 1) (3). Despite this known variability, the Academy of Breastfeeding Medicine (4) suggests that the true range is more likely from 3-20%, indicating that up to one in five mothers can expect to suffer at least one episode of mastitis over the course of their lactation (5).

Whilst an emerging understanding suggests that an underlying cause of mastitis is due to an unbalanced breast milk microbiota (6), a contributing factor in many cases of non-infective and infective mastitis is recognized to be milk stasis (2). Blocked ducts, engorgement and injury to the breast can all result in milk stasis and precipitate non-infective mastitis, whereas cracked nipples and other trauma that disrupt the integrity of the breast may result in infective mastitis (7). Infective mastitis can be caused by bacteria associated with the skin and the infant's mouth. These bacteria are able to gain entry to the breast via cracks in the nipple (8) or by retrograde flow from the nipple back to the alveoli (9). In one study, more women with mastitis were found to have *S. aureus* and Group B streptococci in their milk than those mothers without symptoms (10). Notably, a growing percentage of *S. aureus* infections have been found to be methicillin-resistant (11, 12). In addition, coagulase-negative staphylococci have also been commonly found associated with mastitis (8).

Currently, there is no consensus on the pathophysiology of mastitis, which can be inflammatory, with or without the presence of infection (4). Given the lack of consensus, studies have looked to define predisposing factors and areas of risk, in an effort to provide mothers with information to minimize their likelihood of developing mastitis. In this regard, the use of a breast pump has been, in recent years, associated with a reported increased risk for the development of mastitis (13). Therefore, it is important to outline the role that the breast pump plays in the pathophysiology of mastitis in women so that policy makers and institutions alike can provide mothers and clinicians with up-to-date and appropriate support.

## Predisposing Factors and Treatment

The risk and predisposing factors for mastitis are many and varied although the evidence for their association is weak (4). Of these, it can be noted that the majority are situations that promote or result in the increased likelihood of milk stasis, with the exception of damaged nipples, which is more associated with direct infection. In these situations, milk stasis results in the development of an inflammatory response (14) or provides the time and environment for the growth of pathogenic bacteria. Abou-Dakn and coworkers (15) suggest that measurement of leucocytes and pathogenic bacteria in the milk can be used to differentiate the two causes, although this approach requires the availability of laboratory resources.

Treatment options nearly always focus on the optimization of milk removal with methods to promote the frequent and effective milk removal suggested as a first line, conservative management strategy (4, 15). These care options include ensuring effective and comfortable positioning and latch during breastfeeding whilst paying attention to breastfeeding positions. The incorporation of massage is also recommended. The expression of milk remaining in the breast by the use of hand expression or a breast pump is also suggested and may augment breast drainage and decrease the time to resolution (4). The warming of the breast by wrapping in a warm, moist towel or by taking a warm bath or shower, can activate the oxytocin reflex and promote breast milk flow, whereas the application of cold after milk expression may provide an analgesic/anti inflammatory effect. Pharmacologic management is suggested for cases that do not resolve conservatively, if infectious mastitis is confirmed, or if clinical conditions do not suggest delay (4, 15). In these instances, amoxicillin-clavulanate, dicloxacillin and flucloxacillin, as well as cephalexin (if penicillin intolerance) or clarithromycin (if beta-lactam allergy) are the empiric therapy of choice and are frequently prescribed (4, 16). More recently, the prophylactic supplementation of the mother with a specific probiotic during pregnancy and lactation has also shown to reduce the risk of mothers developing mastitis (6), although such recommendations might better focus on women with a previous history of lactational mastitis.

## Breast Pump and Breast Milk Expression

Previous studies have implicated the breast pump as a potential contributor to the condition of lactational mastitis. Foxman and colleagues (17) reported that for women without a history of mastitis, using a manual breast pump increased the risk of mastitis by 2.1 times, although interestingly, there was no association in women with a history of mastitis. These data are at odds with the perception that the breast pump is a causative factor in the development of mastitis, given that a history of mastitis, itself, is associated with an elevated odds ratio for the development of the condition (17). It would, therefore, be expected that the use of the pump would have been a greater risk factor in this population as well, if it was a causative element for the pathophysiology of mastitis.

**Figure 2:**
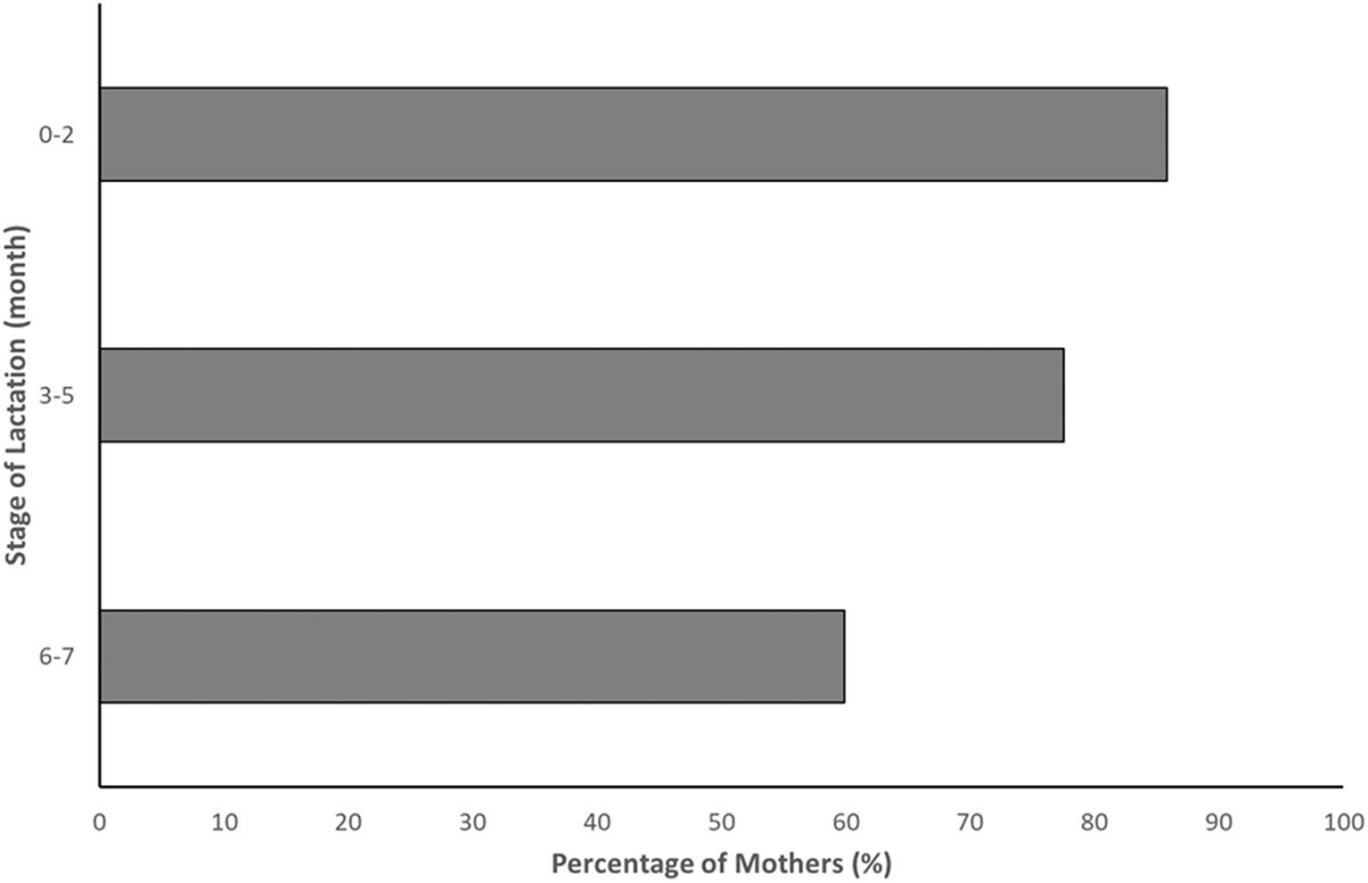
Percent of mothers who pumped or tried to pump milk by infant age, among mothers who breastfed at each age [Data adapted from Centers for Disease Control and Prevention. Results: Breastfeeding and Infant Feeding Practices (19)].

It is also of interest to note that the incidence density for mastitis (Figure 1) shows an increase in the likelihood of mastitis in the early, puerperal, stages of lactation. Breast pump usage does occur in the first four weeks postpartum (18) and remains quite high over the first 6 months of lactation (Figure 2) (19). Despite this, the incidence density for mastitis from various studies published in the literature tends to decrease as stage of lactation progresses (3). Although circumstantial and even when accounting for the decrease in the percentage of mothers breastfeeding over the first six months of lactation, it would be expected that if the breast pump was a causative factor for the development of mastitis the incidence rate would remain high throughout the duration of lactation, following breast pump usage rates.

Another report, by Mediano and coworkers (20), also noted that the use of a breast pump was associated with mastitis, however, the authors went on to suggest that this may be a consequence rather than a cause. Indeed, it was also noted that those studies that had observed a relationship between breast pump usage and the incidence of mastitis used retrospective data pooling, thus making it unclear if breast pump usage was a cause or a consequence of mastitis (21). In this connection, it was suggested that expressing milk by hand or by pump actually reduced the risk of mastitis and was therefore a useful technique for the prevention of mastitis during episodes of oversupply (22). This position is further bolstered by the frequent protocol recommendation to promote breast drainage via the use of hand expression or a breast pump (4, 15, 23). As such, the apparent reverse causation was considered more likely than a direct association, due to the confounding provided by the increased use of the breast pump by the mother with nipple damage when caused as a consequence of breastfeeding (3). This has been further supported by more recent studies finding no statistically significant correlation between the usage of a breast pump and acute mastitis (24, 25).

Lastly, in a study analyzing 1,844 breast pump using mothers (13) who contributed to the Infant Feeding Practices II survey (26), it was suggested that mothers could reduce their risks of problems and injury by choosing breast pumps of better quality and by learning breast pump skills from a person rather than following written or video instructions. In particular, skilled support for breast shield sizing is recommended in order not to negatively impact the amount of breast milk expressed (27). The above data by Qi and coworkers also showed that for mothers using a breast pump, approximately 15% cited any pump-related injury, with only 2.2 % indicating nipple injury, leading to only 0.3% reporting any infection. These data, nevertheless, are below the recognized incidence rate for nipple trauma in the breastfeeding population, which is reported as varying between 29 and 76% (28), suggesting that an appropriate pump usage does not increase the risk of nipple trauma and further supporting the reverse causality proposed by Wilson and coworkers (3).

## The Effect of Vacuum on Milk Removal

**Figure 3:**
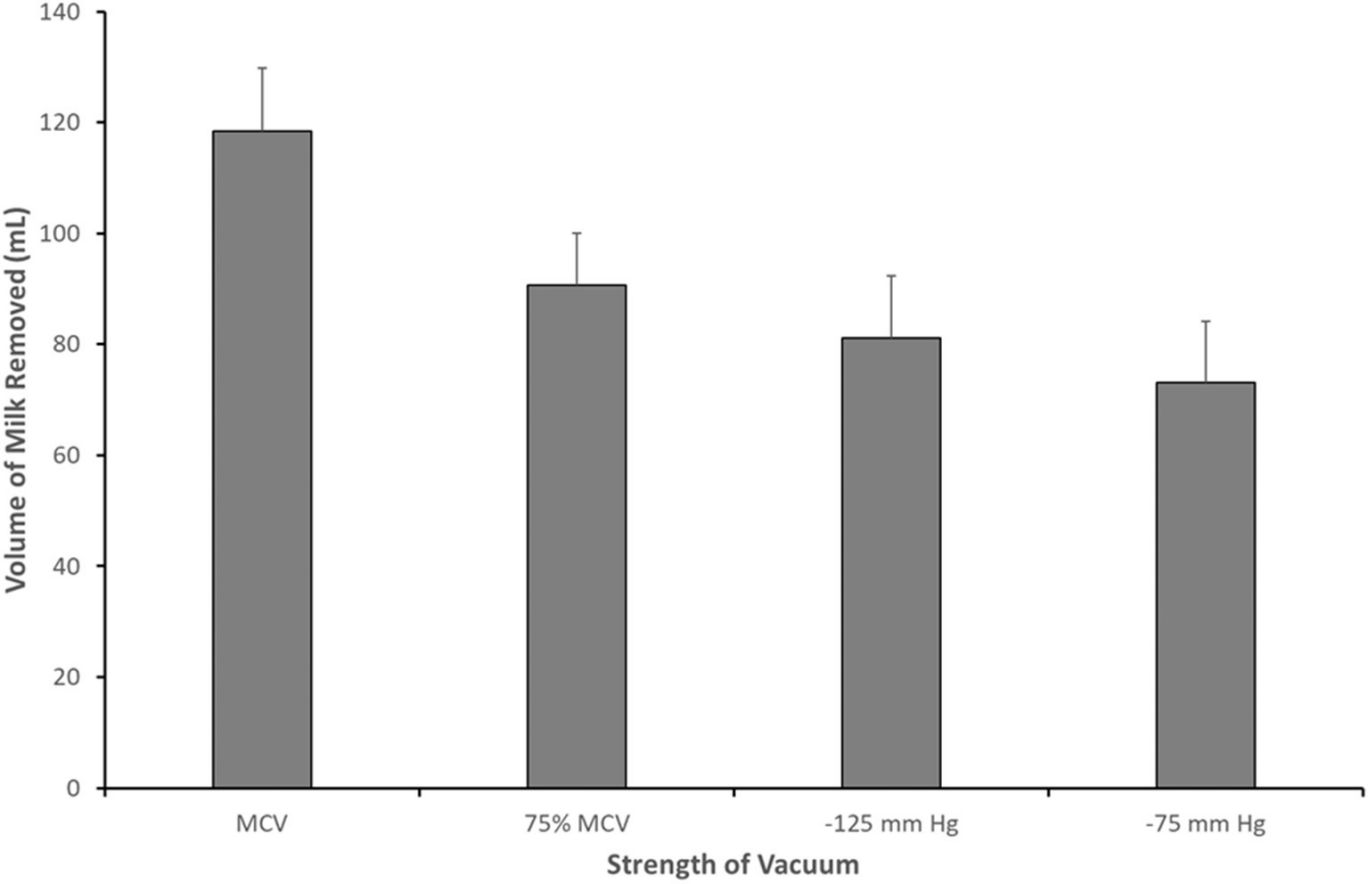
Volume of milk removed when pumping at maximum comfortable vacuum (MCV; −190.7 ± 8.0 mm Hg), 75% of MCV (−143.0 ± 8.8 mm Hg), −125 and −75 mm Hg [Data adapted from Kent et al. (30)].

One area targeted in the implication of a breast pump being a causative factor in the pathophysiology of mastitis has been low vacuum, in particular with respect to the potential poor vacuum performance of a misused or faulty breast pump and the ensuing poor milk removal. The connection between vacuum and milk removal is well established with research on the breastfeeding infant showing that vacuum is, indeed, the main driver of milk removal (29). Furthermore, the relationship between strength of vacuum and milk removal has been extended to the mother using the breast pump (30). This has led to the development of protocols that allow the mother using the breast pump to define her individual maximum comfortable vacuum and then subsequently to use this vacuum setting for all expression episodes (30). Whilst this practice will optimize milk removal, it should be noted that a reduction in vacuum of the breast pump did not result in the complete failure of the breast pump to remove any milk (Figure 3).

These data suggest that even with less than optimal vacuum, some milk removal is achieved. Indeed, with vacuums as weak as −75 mm Hg, Kent and colleagues (30) observed mothers removing, on average, 73.9 mL, suggesting that some milk drainage is achievable when vacuum is low and that complete milk stasis is avoided.

Despite the observation that even low vacuum will result in some milk removal, mothers should be provided anticipatory guidance on hand expression techniques and/or the optimal use of a breast pump as a critical management step in the treatment of mastitis is the frequent and effective removal of milk (4). With regard to the use of a breast pump, this includes practical aspects such as actual pump selection, relative to maternal needs, as well as evidence based characteristics specific to optimizing individualized pump performance (31, 32). These include the use of correctly sized breast shields (27), double pumping (33), when possible and the determination of the mother's maximum comfortable vacuum (30).

## Discussion

It is important to note that with the key role of milk stasis acknowledged as a driver for the pathophysiology of mastitis, the suggested use of the breast pump has become a complementary strategy of the conservative management approach (4, 15, 34). In situations of poor infant positioning and attachment that remain unresponsive to skilled breastfeeding counseling, the inefficient drainage of the breast by the breastfeeding infant can be overcome with the use of hand expression and/or, particularly when the expression is required for a prolonged time, the breast pump, facilitating milk removal and hastening the resolution of the problem (4). Furthermore, in situations of extreme nipple pain from direct breastfeeding, the proper use of hand and/or breast pump expression, in order not to create or amplify any nipple issues, can prove to be less painful (35), and provides the mother with another option to maintain milk removal as the core issue of nipple pain is being resolved.

Given the emerging evidence in the literature regarding the underlying role of mammary gland microbiota dysbiosis in the development of mastitis (6), these data rather suggest that the use of a breast pump is unlikely to be a driver of the pathophysiology of mastitis in women and that the associations presented in the literature are most likely due to reverse causation. In support of this is the decreasing incidence density of mastitis over the course of lactation, despite breast pump usage remaining relatively high over the same timeframe. Moreover, one of the major pathways in the development of mastitis, milk stasis, has been shown to be avoided even in situations of low pump vacuum, as a result of the choice of the mother or in the case of pump misuse. In this connection, in addition to hand expression, the breast pump, when used appropriately in terms of indication and correctness of use, should be considered a valuable tool in the treatment of mastitis, facilitating the drainage of the breast when the mother is unable to breastfeed.

## Data Availability Statement

The original contributions presented in the study are included in the article/supplementary material, further inquiries can be directed to the corresponding author.

## Author Contributions

LRM conceptualized and drafted the initial manuscript. LRM and RD reviewed and edited the manuscript. Both authors contributed to the article and approved the submitted version.

## Conflict of Interest

LRM is an employee of Medela AG, Switzerland. The remaining author declares that the research was conducted in the absence of any commercial or financial relationships that could be construed as a potential conflict of interest.

## Publisher's Note

All claims expressed in this article are solely those of the authors and do not necessarily represent those of their affiliated organizations, or those of the publisher, the editors and the reviewers. Any product that may be evaluated in this article, or claim that may be made by its manufacturer, is not guaranteed or endorsed by the publisher.
